# DNA Topoisomerases Maintain Promoters in a State Competent for Transcriptional Activation in *Saccharomyces cerevisiae*

**DOI:** 10.1371/journal.pgen.1003128

**Published:** 2012-12-20

**Authors:** Jakob Madsen Pedersen, Jacob Fredsoe, Morten Roedgaard, Lotte Andreasen, Kamilla Mundbjerg, Mogens Kruhøffer, Marie Brinch, Mikkel Heide Schierup, Lotte Bjergbaek, Anni Hangaard Andersen

**Affiliations:** 1 Laboratory of Genome Research, Department of Molecular Biology and Genetics, Aarhus University, Aarhus, Denmark; 2 Aarhus University Hospital, Skejby, Aarhus, Denmark; 3 Bioinformatics Research Center, Department of Biology, Aarhus University, Aarhus, Denmark; State University of New York at Stony Brook, United States of America

## Abstract

To investigate the role of DNA topoisomerases in transcription, we have studied global gene expression in *Saccharomyces cerevisiae* cells deficient for topoisomerases I and II and performed single-gene analyses to support our findings. The genome-wide studies show a general transcriptional down-regulation upon lack of the enzymes, which correlates with gene activity but not gene length. Furthermore, our data reveal a distinct subclass of genes with a strong requirement for topoisomerases. These genes are characterized by high transcriptional plasticity, chromatin regulation, TATA box presence, and enrichment of a nucleosome at a critical position in the promoter region, in line with a repressible/inducible mode of regulation. Single-gene studies with a range of genes belonging to this group demonstrate that topoisomerases play an important role during activation of these genes. Subsequent in-depth analysis of the inducible *PHO5* gene reveals that topoisomerases are essential for binding of the Pho4p transcription factor to the *PHO5* promoter, which is required for promoter nucleosome removal during activation. In contrast, topoisomerases are dispensable for constitutive transcription initiation and elongation of *PHO5*, as well as the nuclear entrance of Pho4p. Finally, we provide evidence that topoisomerases are required to maintain the *PHO5* promoter in a superhelical state, which is competent for proper activation. In conclusion, our results reveal a hitherto unknown function of topoisomerases during transcriptional activation of genes with a repressible/inducible mode of regulation.

## Author Summary

Gene expression is controlled at many different levels to assure appropriate responses to internal and environmental changes. The effect of topological changes in the DNA double helix on gene transcription *in vivo* is a poorly understood factor in the regulation of eukaryotic gene expression. Topological changes are constantly generated by DNA tracking processes and may influence gene expression if not constantly removed by DNA topoisomerases. For decades it has been generally accepted that these enzymes regulate transcription by removing excess topological strain generated during tracking of the RNA polymerase, but we still lack a more holistic view of how these enzymes influence gene transcription in their native environment. Here, we examine both global and gene-specific changes in transcription following lack of DNA topoisomerases in budding yeast. Taken together, our findings show that topoisomerases play a profound role during transcriptional activation of genes with a repressible/inducible mode of regulation. For the *PHO5* gene, which is investigated in more detail, we demonstrate that topoisomerases are required for binding of a transcription factor, which is crucial for promoter opening during *PHO5* activation. Our data thus suggest that inducible gene promoters are highly sensitive to changes in DNA superhelicity.

## Introduction

Early studies of transcription have demonstrated that DNA topoisomerases are important in the transcription process [1]. The enzymes transiently break and rejoin the phosphodiester backbone of DNA to allow the passage of individual DNA strands or double helices through one another [2], [3]. In this way they regulate DNA superhelicity and solve topological problems arising during DNA metabolism. In *Saccharomyces cerevisiae*, DNA superhelicity is influenced by topoisomerases I and II (Top1p and Top2p), encoded by the *TOP1* and *TOP2* genes, respectively [3]. Although both enzymes are able to relax supercoiled DNA, they show different substrate preferences, with Top2p being much faster than Top1p, when nucleosomal DNA is relaxed, whereas the opposite is the case during relaxation of naked DNA [4]. Despite these differences, early studies in yeast have demonstrated that transcription is more or less unaffected in yeast cells lacking either Top1p or Top2p, indicating that the two enzymes are redundant in the transcription process. Conversely, *top1Δtop2ts* mutants grown under restrictive conditions display a decreased rate of both rRNA and mRNA synthesis [1].

Transcription and DNA supercoiling are linked by a cause-effect relationship that operates in both directions. The transcriptional effect on supercoiling is explained by the Twin-Supercoiled-Domain-Model, which predicts that two domains of DNA supercoiling are generated during transcription elongation, provided that the RNA polymerase cannot rotate freely around the template, and that DNA rotation is hindered [5]. Thus, positive and negative supercoiling will be formed in front of and behind the advancing polymerase, respectively. The model, which has gained support from both *in vitro* and *in vivo* studies [1], [6], [7], implies that a gradient of positive and negative supercoils will dissipate from an active transcription unit if topoisomerase activity is lacking. The effect exerted by supercoiling on transcription has in many cases been demonstrated to depend on the sign of the supercoils. Thus, positive supercoiling has been suggested to impair transcription initiation as well as elongation by inhibition of strand separation [8], [9]. In contrast, negative supercoiling has been suggested to be more favorable for transcription, in that it may facilitate transcription initiation by enhancing complex formation at promoters [10]–[12].

The crosstalk between DNA supercoiling and transcription still remains elusive *in vivo*, where chromatin structure adds another layer of complexity. Dissociation and re-association of nucleosomes will release and absorb negative superhelicity, respectively, with a potential impact on transcription [13], and topoisomerases have indeed been demonstrated to affect nucleosome dynamics [14]–[16]. Furthermore, chromatin has been suggested to adapt to positive supercoiling by a slight conformational change, which is reverted upon relaxation by either Top1p or Top2p [4]. This implies that the chromatin fiber is a torsionally resilient structure, which can act as a topological buffer *in vivo* and facilitate dissipation of topological strain [4], [9], [17]. In eukaryotes, a change in DNA superhelicity may thus exert an additional effect on transcription via changes at the chromatin level.

Several studies have suggested that the individual topoisomerases play a role during transcription initiation. Thus, human topoisomerase I has been demonstrated to affect transcription initiation from TATA-containing promoters, functioning as a repressor of basal transcription but as an enhancer of activated transcription [18]. In line with this, studies with yeast Top1p have suggested that the enzyme exerts an inhibitory effect on transcription initiation of a subset of stress-inducible genes located in the silenced subtelomeric regions [19]. Concerning topoisomerase II, experiments performed with a topoisomerase II inhibitor have demonstrated a role of this enzyme in the activation of specific oncogenes, where activation reflects a change in promoter structure [20]. In addition, mammalian topoisomerase IIβ has been found to directly affect transcription initiation of an inducible gene by creating a specific DNA double strand break in the promoter region allowing nucleosome displacement and downstream protein recruitment [21].

Recent studies of transcription using genome-wide approaches have further substantiated a role of topoisomerases during transcription initiation. In a study performed in *S. pombe*, Top1p was suggested to be directly responsible for nucleosome disassembly in gene promoters prior to transcription [14]. However, in a study performed in *S. cerevisiae*, Top1p and Top2p were suggested to act redundantly to allow recruitment of RNA polymerase II to nucleosome-free promoters rather than to act in nucleosome removal *per se* [22]. In both cases topoisomerases were found to bind preferentially to promoter regions of highly active genes. The precise role of DNA topoisomerases in transcription is thus still not clear. Indeed, steps upstream of the engagement of polymerases and nucleosome removal could be influenced by DNA supercoiling, i.e. binding of transcriptional activators or repressors.

In the present study, we have combined microarray gene expression analyses and single-gene studies using *S. cerevisiae* strains lacking either one or both DNA topoisomerases to unravel the implications of these enzymes on transcription. Although we demonstrate that the requirement for topoisomerases generally correlates with transcriptional activity we find that DNA topoisomerases have a major impact on transcription of a subset of genes, which are not unified by being highly transcribed *per se*. Rather, the most affected genes are characterized by features associated with highly regulated transcription initiation. Studies of several genes from this subgroup demonstrate that topoisomerases indeed are required for adequate and timely transcriptional induction. Finally, in case of the inducible *PHO5* gene, we demonstrate that topoisomerase-mediated relaxation is required for binding of the Pho4p transcription factor, whereas constitutive *PHO5* transcription is unaffected by topoisomerase deficiency.

## Results

### Topoisomerases I and II act redundantly in genome-wide transcription, but global down-regulation occurs in the absence of both enzymes

**Figure 1:**
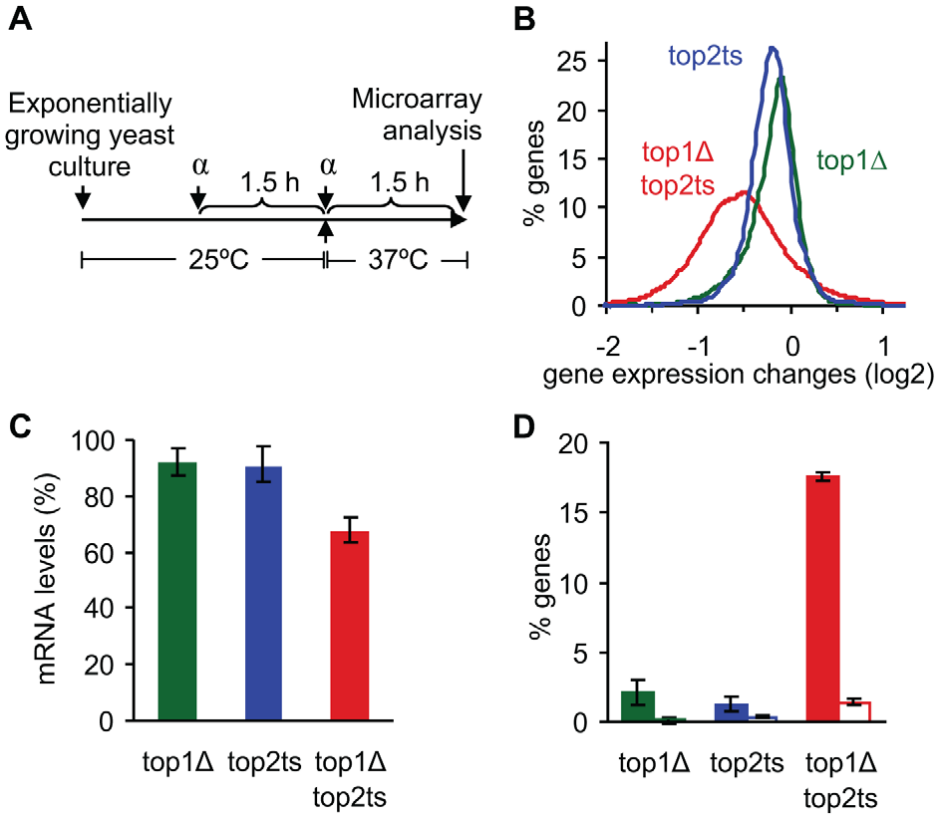
Global reduction in mRNA levels occurs due to lack of topoisomerases I and II. (A) Experimental setup showing the timing of G1 arrest by α-factor (α) and inhibition of Top2p at 37°C. (B) Distribution of gene expression changes (between mutant and wild-type) in topoisomerase single and double mutants. (C) Relative mRNA levels were calculated using the total microarray signal intensities in mutants and wild-type. mRNA levels in wild-type were set to 100%. Error bars represent ± one standard deviation from biological triplicates. (D) Percentage of genes up- and down-regulated 2-fold or more (open and filled columns, respectively). Error bars represent ± one standard error of the means from biological triplicates.

To investigate the impact of DNA topoisomerases I and II (Top1p and Top2p, respectively) on genome-wide transcription, we examined the *S. cerevisiae* polyadenylated transcriptome by microarray analysis in *top1Δ*, *top2ts*, *top1Δtop2ts* and the isogenic wild-type strain. To bypass genome-wide effects of topological challenges caused by replication [23], [24] as well as abortive mitosis due to lack of Top2p activity, the window of transcription was limited to the G1-phase of the cell cycle, and cells were grown at the restrictive temperature for conditional inhibition of Top2p (Figure 1A).

Due to the expected drop in RNA synthesis in cells lacking topoisomerase activity [1], external normalization was used to compensate for unbalanced gene expression changes [25] (see Text S1). As seen in Figure 1B, a genome-wide decrease of most transcripts is observed in *top1Δtop2ts*, reflecting an absolute drop in mRNA abundance at the cellular level of ∼30% (Figure 1C). Furthermore, around 20% of all genes are 2-fold or more up- or down-regulated in the double mutant, where the down-regulated genes account for ∼17% (Figure 1D). In contrast, the single mutants show a drop of only 10% in mRNA abundance (Figure 1C) and a relatively low number of de-regulated genes (Figure 1D), suggesting a redundant nature of the two enzymes in genome-wide transcription as indicated earlier [1].

### Topoisomerase dependency is associated with transcriptional activity but not transcript length

To address, whether the global transcriptional down-regulation in topoisomerase deficient cells can be explained by effects predicted by the Twin-Supercoiled-Domain-Model, we considered two simple parameters, transcriptional activity and transcript length, which are both proportional to the number of DNA supercoils produced during transcription of a specific gene [5].

**Figure 2:**
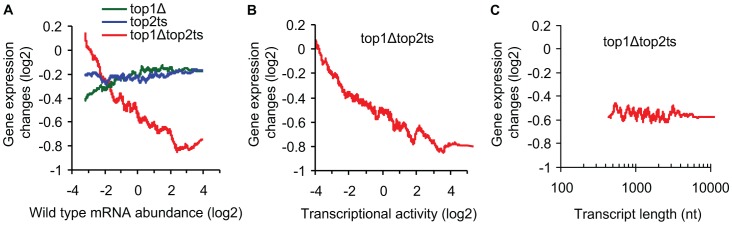
Transcriptional activity and not transcript length reflects topoisomerase dependency. (A) *top1Δ, top2ts*, and *top1Δtop2ts* gene expression changes plotted against mRNA abundance in wild-type cells as a 200 gene moving average. (B) *top1Δtop2ts* gene expression changes plotted against transcriptional activity in wild-type cells as a 200 gene moving average. (C) *top1Δtop2ts* gene expression changes plotted against transcript length [27] as a 200 gene moving average. Nt, nucleotides.

As shown in Figure 2A, a plot of gene expression changes in *top1Δtop2ts* against wild-type mRNA abundances reveals that genes with higher mRNA abundance are more affected by topoisomerase deficiency relative to genes with lower abundance (Pearson correlation = −0.35). In contrast to the double mutant, the single mutants show no correlation between transcript changes and wild-type mRNA abundance, consistent with the redundant nature of the two enzymes in genome-wide transcription.

Relative measures of transcriptional activity for every gene were obtained from measures of mRNA abundance by taking gene specific values of polyA mRNA breakdown into account as described by Schreiber and co-workers [26]. As shown in Figure 2B, we found increasing topoisomerase dependency with increasing transcriptional activity (Pearson correlation = −0.34). We therefore conclude that topoisomerase deficiency generally has a larger impact on highly active genes relative to less active genes. A similar conclusion was reached by use of average RNA polymerase II occupancy instead of transcriptional activity (Figure S1).

We next related the *top1Δtop2ts* transcript changes to a genome-wide survey of transcript lengths [27]. However, no correlation between transcript length and topoisomerase dependency was found (Figure 2C) (Pearson correlation = 0.00). Furthermore, a statistical test of all transcripts shorter than 0.5 kb (n = 355) and larger than 4.5 kb (n = 122) revealed no difference in the distribution of *top1Δtop2ts* gene expression changes (*P* = 0.87, Wilcoxon rank-sum test for different distribution). In conclusion, the data demonstrate that the requirement for topoisomerases during global gene transcription increases with increasing transcriptional activity, but is independent of transcript length.

### Topoisomerases affect transcription of metabolic and stress-related genes as well as genes with a TATA box in the promoter region

Despite the finding that transcriptional activity is a global indicator of topoisomerase dependency, we noticed that a range of the most actively transcribed genes were not among the most de-regulated genes in *top1Δtop2ts* (Figure S2). Thus, features other than transcriptional activity *per se* may be responsible for topoisomerase requirements during gene transcription.

To look for common traits and overrepresentation of biological functions among the genes strongly affected by topoisomerase deficiency, we performed gene ontology analyses. As reported in Table S1, topoisomerase deficiency preferentially affects transcription of genes involved in diverse metabolic pathways and in the response to stress, which are genes for which transcription is typically altered, when environmental conditions are changed [28], [29]. We therefore investigated, whether genes affected by topoisomerases display a higher responsiveness to environmental changes relative to genes, which are unaffected by topoisomerases. A measure for responsiveness to environmental changes was derived for every gene by calculating the average transcript change of the gene across the Gasch data set consisting of 173 microarray transcription profiles obtained from cells subjected to diverse environmental perturbations [28]. As reported in Table S2 we found that topoisomerase dependent genes, including both up- and down-regulated genes, have significantly higher responsiveness to environmental changes relative to the rest of the genome. Our data therefore suggest that DNA topoisomerases play an important role in the regulatory network of gene expression in the response to environmental changes. This finding prompted us to look for denominators that are common to transcription of topoisomerase-dependent genes. Indeed, we found that this group of genes has a significant enrichment of genes with a TATA-box in the promoter region as well as genes dependent on the SAGA (Spt-Ada-Gcn5-acetyltransferase) complex (Table S2, Figure S2). Both are features, which predominantly are associated with regulation of genes with a repressible/inducible mode of regulation [30], [31]. In contrast, TATA-less genes tend to have housekeeping functions and are more constitutively transcribed. Taken together, these analyses suggest that topoisomerase-dependent genes are highly regulated.

### Topoisomerase-dependent genes have high transcriptional plasticity and are governed by chromatin regulation

**Figure 3:**
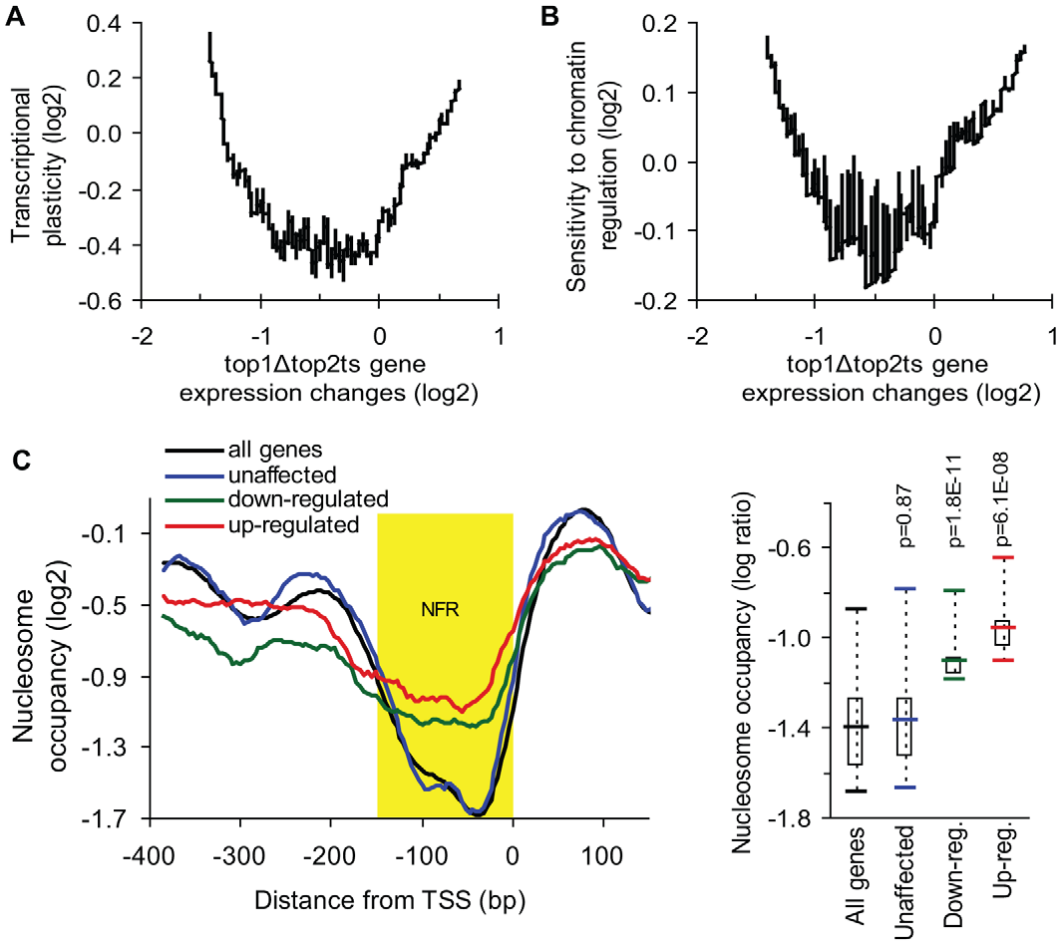
Genes de-regulated in *top1Δtop2ts* have high transcriptional plasticity and are chromatin-regulated. (A) Transcriptional plasticity [32] plotted against *top1Δtop2ts* gene expression changes (200-gene moving average). (B) Sensitivity to chromatin regulation [34] plotted against *top1Δtop2ts* gene expression changes (200-gene moving average). (C) *Left panel*, the average nucleosome-binding pattern around the transcription start site (TSS) was compared between groups of the 100 most unaffected, the 100 most up-regulated, the 100 most down-regulated genes in *top1Δtop2ts*, and the average pattern for all genes in the yeast genome [35]. Nucleosome-free region (NFR) is highlighted in yellow. *Right panel*, statistical analysis of nucleosome occupancy in the NFR displayed by a box plot. *P*-values were calculated by an unpaired, two-sample t-test assuming equal variances.

To investigate if topoisomerase-dependent genes have a higher regulatory capacity relative to genes, which are unaffected by topoisomerase deficiency we took advantage of a gene-specific measure of transcriptional plasticity. This measure has previously been defined as the dynamic range of transcript changes a gene displays in a large collection of >1,500 microarray analyses of gene expression [32]. Intriguingly, when plotting transcriptional plasticity against gene expression changes in *top1Δtop2ts*, we found a curvilinear relationship (Figure 3A). This demonstrates that genes strongly affected by topoisomerase deficiency, including both up- and down-regulated genes, display a high transcriptional plasticity relative to less affected genes. Notably, transcriptional plasticity is correlated with topoisomerase dependency independent of expression levels (Figure S3). Overall, the characterization of topoisomerase-dependent genes as being highly regulated across a multitude of conditions suggests that topoisomerase deficiency perturbs some of the regulatory features inherent to this gene class.

Transcription of highly regulated genes is generally associated with chromatin remodeling and histone modifying activities, which regulate the access of transcription factors and the general transcription machinery to promoters [33]. To further analyze the properties of the topoisomerase-dependent genes, we therefore used a measure of the sensitivity to chromatin regulation, which has been calculated as the expression variability a gene displays in 141 microarray profiles obtained in the absence of different chromatin modifiers [34]. This measure was plotted against gene expression changes in *top1Δtop2ts* (Figure 3B). As shown in Figure 3B, the genes, which are most affected by topoisomerase deficiency, show the highest sensitivity to chromatin regulation in accordance with the high transcriptional plasticity of these genes. Thus, genes which are activated or repressed at the chromatin level are prone to be influenced by DNA topoisomerases. To further support this finding we calculated pairwise Pearson correlations between the *top1Δtop2ts* transcription profile and the profiles from more than 1,000 different microarrays from yeast. In this screen the most significant correlations were found to transcription profiles generated from yeast strains lacking factors affecting regulation of transcription via chromatin (e.g. *spt6ts*, *spt16ts*, *gcn5* mutation, histone depletion and histone tail deletion, Figure S4).

The observed correlation between de-regulated transcription in *top1Δtop2ts* and measures of transcriptional plasticity and chromatin regulation encouraged us to address, whether the genes with the strongest dependency on topoisomerases have a different promoter chromatin architecture compared to topoisomerase-independent genes. We therefore used a map of nucleosome occupancy across the yeast genome [35] to examine the nucleosome binding pattern in the promoter region of these genes. The 100 most up- and down-regulated genes as well as the 100 most unaffected genes in *top1Δtop2ts* were selected to identify a possible topoisomerase-dependent promoter nucleosome architecture. As seen in Figure 3C and Figure S5, genes, which are strongly affected by topoisomerase deficiency, have a significant higher nucleosome occupancy in the conserved nucleosome-free region, which is a region known to be enriched for binding of transcription factors and chromatin regulators influencing transcription initiation [36].

Taken together, the data from the genome-wide analyses suggest that topoisomerase deficiency affects transcription of a group of genes, which can be characterized as being highly regulated, thus having a repressible/inducible mode of regulation. Given that highly regulated genes are characterized by tight control of initiation rather than elongation, the data point to an important role of topoisomerases during transcription initiation.

### Topoisomerases are needed during transcription of a range of inducible genes

**Figure 4:**
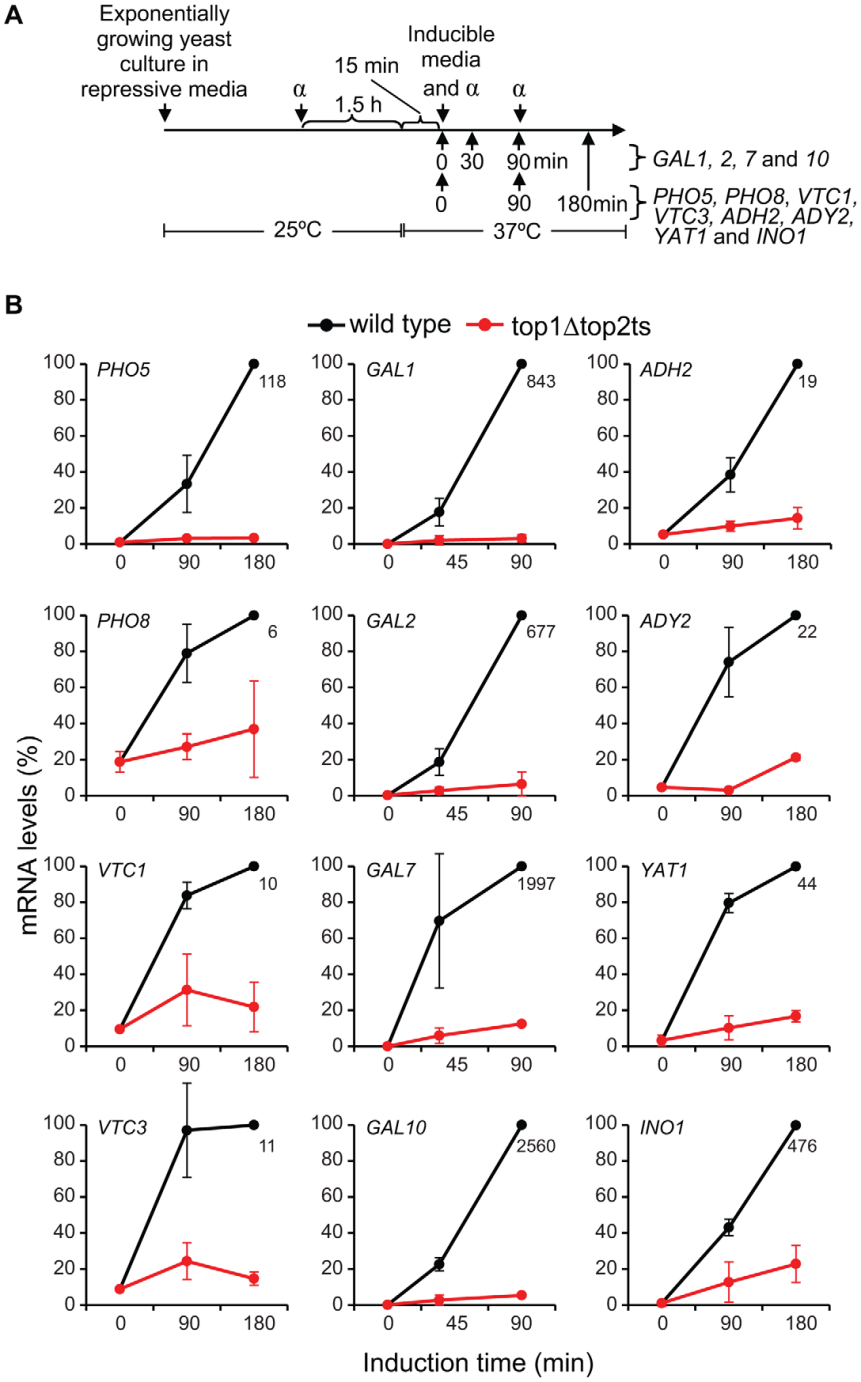
Topoisomerases are required for transcriptional induction of a range of inducible genes. (A) Experimental setup. α indicates α-factor. (B) Time-course experiments of induced gene expression in wild-type and *top1Δtop2ts* cells. The mRNA levels of the indicated genes were quantified by qPCR at the indicated time points after transfer of cells to inducible conditions and normalized to the mRNA level obtained in the wild-type at the latest time point (set to 100%). Averages from two individual experiments are shown with error bars representing ± one standard deviation. Numbers indicate the mean fold increase in wild-type cells at the latest time point.

We next wanted to substantiate the genome-wide findings by analyses of specific genes with a repressible/inducible mode of regulation that are known to be environmentally regulated and dependent on chromatin structure. For this purpose, twelve genes were selected from four commonly studied gene systems, representing phosphate- (*PHO5*, *PHO8, VTC1, VTC3*), galactose- (*GAL1, GAL2, GAL7, GAL10*), glucose- (*ADH2, ADY2, YAT1*) and inositol-regulated (*INO1*) promoters. In order to study the induction capabilities, wild-type and *top1Δtop2ts* cells were cultured under repressive conditions and transferred to the respective inducible conditions (see Text S1) as outlined in the experimental setup presented in Figure 4A. As demonstrated in Figure 4B, transcription of all twelve genes is significantly compromised in the absence of DNA topoisomerases. Albeit the selected genes have important differences in many aspects associated with regulation of transcription, e.g. activator and co-factor requirements, the data show that topoisomerases have comparable transcriptional effects on the different inducible gene systems. To verify that topoisomerase deficiency exerts a specific effect on transcription of inducible genes, and furthermore confirm the specificity of the different inducible conditions, we included three housekeeping genes, *ESC1*, *ACT1*, and *GAPDH*, where measurements of transcript accumulation were obtained under either phosphate-, galactose-, or glucose-inducible conditions (Figure S6). These genes showed virtually no transcript changes in wild-type and *top1Δtop2ts* cells in the time frame, where the inducible genes showed up to several thousand fold increase in mRNA levels in the wild-type. Taken together, our results support a model, where topoisomerases are needed for adequate transcription of regulated genes, and thus corroborate our findings from the microarray gene expression analysis.

### Topoisomerases are required for transcriptional activation of *PHO5* but become dispensable once the gene is activated

**Figure 5:**
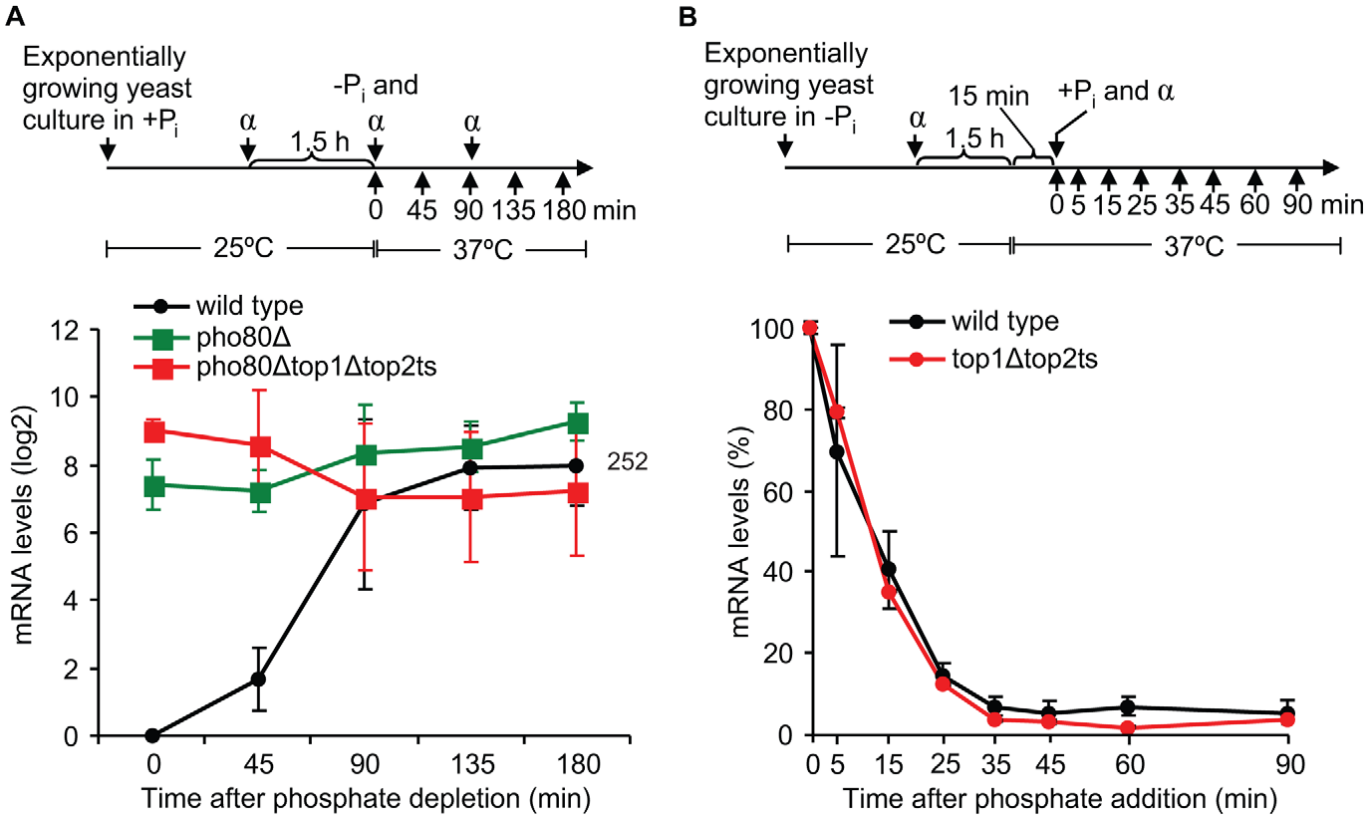
Topoisomerases are dispensable for transcription once the *PHO5* promoter is activated, and they are not required during *PHO5* inactivation. (A) Time-course experiments of *PHO5* transcription in wild-type, *pho80Δ*, and *pho80Δtop1Δtop2ts* cells after transfer from high phosphate to phosphate-free conditions. *Upper panel*, experimental setup. *Lower panel*, the *PHO5* mRNA levels were quantified at the indicated time points, normalized to the wild-type level at the 0 min time point (set to 1) and presented on a log2-scale. Number indicates the mean fold increase in wild-type cells at the latest time point. (B) Time course experiment of *PHO5* transcriptional inactivation in wild-type and *top1Δtop2ts* cells. *Upper panel*, experimental setup. *Lower panel*, the *PHO5* mRNA levels were quantified at the indicated time points and normalized to the level at the 0 min time point (set to 100%). In A and B the averages from three and two individual experiments, respectively, are shown. Error bars represent ± one standard deviation. α indicates α-factor and +P_i_ and -P_i_ indicate high and no phosphate, respectively.

To investigate in which step during transcription of a regulated gene topoisomerases exert their function, we focused on the well-characterized *PHO5* gene, which was found to absolutely require topoisomerases as demonstrated in Figure 4B. This gene is repressed under high phosphate conditions, where the transcription factor Pho4p is phosphorylated by the Pho80p/Pho85p complex and retained in the cytoplasm. In the un-phosphorylated state under phosphate-free conditions, Pho4p enters the nucleus, where it binds the *PHO5* promoter and trans-activates chromatin remodeling, thus being essential for *PHO5* induction by promoter nucleosome removal [37], [38]. To initially determine if topoisomerases are required for *PHO5* activation *per se* or for continued *PHO5* transcription upon activation, we took advantage of the fact that deletion of *PHO80* leads to constitutive expression of *PHO5* regardless of phosphate conditions [39]. Wild-type cells as well as *pho80Δ* and *pho80Δtop1Δtop2ts* mutants were analyzed in parallel for accumulation of *PHO5* transcripts. As expected, both mutants show high *PHO5* transcription levels under high phosphate conditions at the non-restrictive temperature (0 min time point), where the wild-type cells are fully repressed (Figure 5A). However, upon transfer to inducible conditions at the restrictive temperature, *pho80Δtop1Δtop2ts* still accumulates *PHO5* mRNA at a level comparable to *pho80Δ* and similar to the transcription level from the fully active *PHO5* promoter in wild-type cells. The result demonstrates that topoisomerases have no effect on transcription from an already activated *PHO5* promoter, and we therefore conclude that topoisomerases are needed for activation of *PHO5* but not for continuous transcription initiation and elongation.

To investigate if topoisomerases also play a role during transcriptional inactivation of *PHO5*, we performed an experiment, where wild-type and *top1Δtop2ts* cells were grown under inducible conditions and then transferred to high phosphate to shut down expression. As seen in Figure 5B, the kinetics in the decrease of *PHO5* mRNA levels were equivalent in *top1Δtop2ts* and wild-type cells, strongly indicating that topoisomerases are dispensable during transcriptional repression of *PHO5*. Thus, although topoisomerases are essential for *PHO5* activation, they do not seem to be required during *PHO5* inactivation.

### Transcription of *PHO5* is supercoiling sensitive

**Figure 6:**
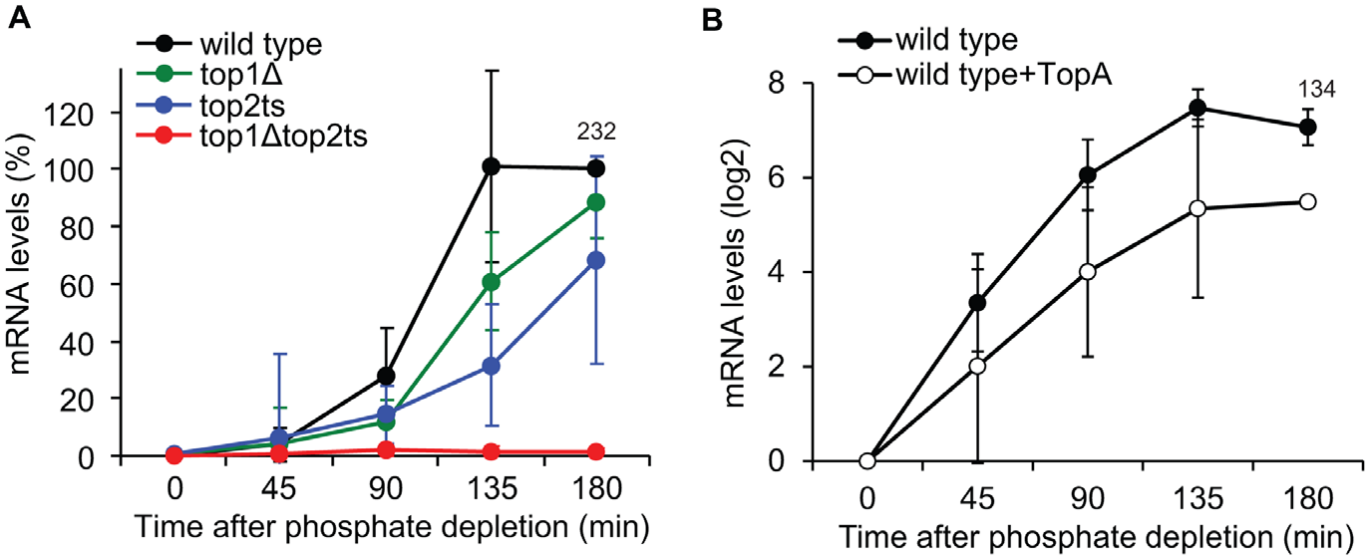
Changes in global DNA supercoiling levels affect *PHO5* transcription. (A) Time-course experiment of *PHO5* transcriptional activation in wild-type, *top1Δ*, *top2ts*, and *top1Δtop2ts* cells. The experimental setup was as described for Figure 5A. The quantified *PHO5* mRNA levels were normalized to the wild-type level at the 180 min time point (set to 100%). (B) Time course of *PHO5* transcriptional activation in wild-type cells with and without expression of TopA from a high-copy YEp-TopA plasmid [8]. The experimental setup was as described for Figure 5A except for TopA expression. The *PHO5* mRNA levels were quantified at the indicated time points, normalized to the mRNA level at the 0 min time point (set to 1) and presented on a log2-scale. In A and B averages from three individual experiments are shown with error bars representing ± one standard deviation. Numbers indicate the mean fold increase in wild-type cells at the latest time point.

To address how DNA topoisomerases affect transcriptional activation of *PHO5*, we first compared the accumulation of *PHO5* mRNA levels in the topoisomerase single and double mutants (Figure 6A). The analysis shows that, whereas wild-type cells reach full induction after approximately 135 min under phosphate-free conditions, lack of either Top1p or Top2p results in a kinetic delay in *PHO5* mRNA accumulation. In contrast, complete lack of topoisomerase activity results in a synthetic phenotype with an absolute inhibition of *PHO5* activation. The fact that *PHO5* transcription is sensitive to topoisomerase dosage strongly suggests that it is the total relaxation capacity of the cell, which is important for *PHO5* transcription. The result thus indicates that *PHO5* activation is influenced by changes in DNA superhelicity.

To further investigate this we took advantage of the *E. coli* DNA topoisomerase I enzyme (TopA). This enzyme only relaxes negative supercoiling and has earlier been used to alter DNA superhelicity on a global scale [8], [9]. As shown in Figure 6B, expression of TopA from a high-copy plasmid in wild-type cells leads to reductions in *PHO5* transcript levels upon transfer to inducing conditions, strongly suggesting that *PHO5* activation is supercoiling sensitive.

### Topoisomerases are required for Pho4p binding prior to promoter nucleosome removal during *PHO5* activation

**Figure 7:**
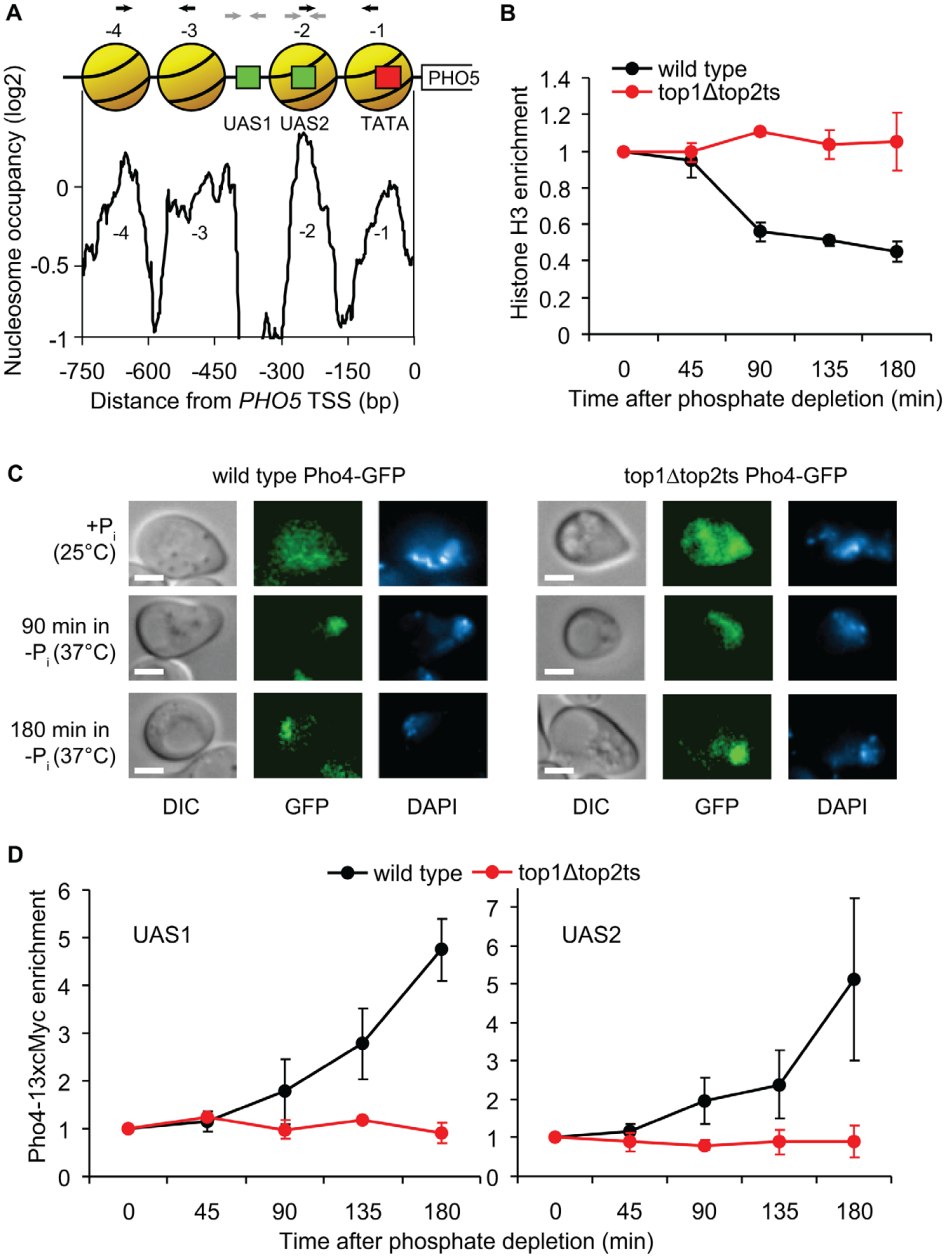
Topoisomerases are essential for binding of the Pho4p transcription factor. (A) Nucleosome occupancy profile of the *PHO5* promoter [35]. Positioned nucleosomes (yellow), which are removed upon *PHO5* induction, are denoted −1 to −4 relative to the transcription start site (TSS). Red box indicates TATA box and green boxes indicate upstream activating sequences, UAS1 and UAS2, which both contain a Pho4p binding site. Black and grey arrows indicate the primers used in the H3 and Pho4-13xcMyc ChIP experiments, respectively. (B) Time course ChIP analysis of nucleosome removal from the *PHO5* promoter in wild-type and *top1Δtop2ts* cells following transcriptional activation. Experimental setup was as described for Figure 5A. The plot depicts average levels of histone H3 in nucleosome regions −1 to −2 and −3 to −4, respectively, in the *PHO5* promoter. H3 binding levels were normalized relative to the binding under un-induced conditions at the 0 min time point (set to 1). (C) Localization of Pho4-GFP was investigated by fluorescence microscopy in wild-type and *top1Δtop2ts* cells grown under high phosphate conditions (+P_i_) at 25°C, and 90 and 180 min after shifting cells to phosphate-free medium (−P_i_) at 37°C for inhibition of Top2p. The experimental setup was as described for Figure 5A. Differential interference contrast (DIC) and fluorescence (GFP) images of representative cells are shown, and the nuclei are indicated by DAPI (4′,6-diamidino-2-phenylindole) staining of DNA. Scale bars represent 2 µm. (D) Time course ChIP analyses of Pho4-13xcMyc recruitment kinetics in the *PHO5* promoter in wild-type and *top1Δtop2ts* cells following transcriptional activation. The experimental setup was as described for Figure 5A. The plots depict levels of Pho4-13xcMyc binding to UAS1 (*left panel*) and UAS2 (*right panel*) in the *PHO5* promoter, although the resolution of the ChIP assay may be insufficient to discriminate between the two sites, and Pho4p binding at the low affinity UAS1 site may be below the detection threshold of the assay. Pho4-13xcMyc binding levels were normalized relative to the binding under un-induced conditions at the 0 min time point (set to 1). In B and D averages from three individual experiments are shown and error bars represent ± one standard deviation.

To examine the underlying cause of the perturbed *PHO5* activation in *top1Δtop2ts* cells, we next studied the impact of topoisomerase deficiency on regulation of crucial steps upstream of transcription initiation. A previous study has shown an absolute requirement for nucleosome removal *in trans* from the *PHO5* promoter region for transcription initiation to occur, which is dependent on binding of the Pho4p transcription factor to the *PHO5* promoter [40]. Figure 7A shows the promoter structure of *PHO5* with a nucleosome map, illustrating Pho4p binding sites and four highly positioned nucleosomes covering the promoter region.

To investigate if nucleosome removal is affected in *top1Δtop2ts* we used Chromatin immunoprecipitation (ChIP) with an antibody against histone H3 to measure nucleosome occupancy in the *PHO5* promoter in wild-type and *top1Δtop2ts* cells during *PHO5* induction. As shown in Figure 7B, a decrease in the relative amount of qPCR products corresponding to the promoter region with the four nucleosomes is seen with increasing time following induction in the wild-type cells, consistent with results from Hörz and coworkers [41]. In contrast, no decrease is seen in *top1Δtop2ts*. These results suggest that topoisomerases are either required directly for the removal of repressive nucleosomes from the *PHO5* promoter or for a step prior to this activity. We reason that this step cannot be de-regulation of a chromatin remodeling factor as strains disrupted for such factors have been shown to merely give rise to a kinetic delay in *PHO5* activation and not an absolute inhibition [42]–[45]. Although less likely, we can however not rule out that two or more chromatin remodeling factors are de-regulated and together exert a synthetic phenotype.

To eliminate the possibility that defective *PHO5* induction in *top1Δtop2ts* is indirectly caused by disruption of the physiological stimulus leading to transcriptional activation, we analyzed the cellular localization of GFP-tagged Pho4p in wild-type and *top1Δtop2ts* cells. As expected, both strains exhibit nuclear accumulation of Pho4-GFP after 90 and 180 min under phosphate-free conditions at the restrictive temperature (Figure 7C). The results thus limit the window of topoisomerase requirement to a step between Pho4p nuclear entrance and promoter nucleosome removal.

We therefore finally addressed the possibility that binding of Pho4p, which has two binding sites in the *PHO5* promoter (the low affinity UAS1 site and the high affinity UAS2 site) (Figure 7A) is perturbed in *top1Δtop2ts*. We constructed wild-type and *top1Δtop2ts* strains with a 13xcMyc-tagged version of Pho4p (displaying normal *PHO5* induction kinetics, Figure S7), and performed ChIP analyses with a cMyc-antibody to monitor Pho4p binding to the *PHO5* promoter. Intriguingly, in contrast to the situation in wild-type cells, Pho4-13xcMyc is not enriched in the *PHO5* UAS1 and UAS2 regions in *top1Δtop2ts* after transfer of cells to inducible conditions (Figure 7D). Even after 3 h in phosphate-free medium, where *PHO5* is strongly induced in wild-type cells, Pho4p binding levels in the *PHO5* promoter in *top1Δtop2ts* are similar to binding levels in the repressed state. We therefore conclude that topoisomerases are required to allow binding of Pho4p to the *PHO5* promoter during transcriptional activation.

## Discussion

The global gene transcription analyses of budding yeast cells deficient for topoisomerases I and II reveal two major effects: (*i*) highly transcribed genes are generally more dependent on topoisomerase activity than poorly transcribed genes, and (*ii*) genes strongly dependent on topoisomerases are characterized as highly regulated and chromatin-dependent with a repressible/inducible mode of regulation. In support of the genome-wide findings our single-gene studies suggest that topoisomerases play a previously unidentified and important role in transcription of highly regulated, inducible genes. Finally, the in-depth analysis of the inducible *PHO5* gene has revealed that topoisomerase activity is required for binding of the Pho4p transcription factor upstream of promoter nucleosome removal. The findings provide novel insight into the role of DNA topoisomerases for *in vivo* transcription.

### Transcriptional activity is a global indicator of topoisomerase dependency

Our transcriptome analysis reveals that topoisomerase deficiency leads to a general down-regulation, giving rise to a reduction in mRNA levels of approximately 30%. Interestingly, transcriptional activity but not transcript length is an important cause of the global down-regulation in *top1Δtop2ts* (Figure 2). Since transcriptional activity reflects both the rate of elongation and initiation, down-regulation in *top1Δtop2ts* with increasing transcriptional activity can be explained by an impairment of elongation and/or initiation. However, most impairments of elongation will lead to increasing down-regulation with increasing transcript length, which we do not see. Our results are therefore most simply explained by an impairment of initiation of highly transcribed genes in the absence of topoisomerases as suggested by Roca et al. [9]. In support of this, global ChIP-chip studies of Top1p and Top2p have demonstrated that the enzymes bind intergenic/promoter regions in the yeast genome [14], [22], [23] in an activity dependent manner [14], [22].

How do topoisomerases influence initiation of highly transcribed genes? The fact that Top1p and Top2p act redundantly during genome-wide transcription (Figure 1 and Figure 2A) speaks against a structural or more specific role of either enzyme and rather suggests that they act via their common relaxation activity. Thus, the contribution of either enzyme alone seems sufficient to remove supercoiling to a degree, which maintains high levels of gene transcription *in vivo*, but when both enzymes are absent, unresolved supercoiling inhibits initiation in an activity dependent manner.

The observation that the superhelical strain generated during RNA polymerase tracking does not result in a length dependent requirement for topoisomerases suggests that the RNA polymerase is able to track against a supercoiling gradient. Alternatively, the supercoils may rapidly dissipate into flanking chromosomal regions, where they may be buffered by chromatin structural transitions as suggested earlier [4], [17], merge with supercoils of opposite sign, or dissipate out of chromosomal ends by rotation of telomeres [9]. Lack of topoisomerase activity during RNA polymerase tracking may indeed enhance the pressure on alternative pathways for supercoil removal, possibly leading to superhelical changes throughout the chromosomes, including gene promoters, where it eventually may influence transcription initiation. However, we cannot rule out that transcription elongation by each polymerase may be affected by the superhelical strain generated ahead of it as suggested by Ekwall and coworkers based on observations in *S. pombe* [14] and recent studies from *S. cerevisiae*, where transcription of long genes was found to be affected exclusively in *top2ts* cells [46]. Our data suggest that any effect on elongation will have to be gene-length independent in *top1Δtop2ts* cells (Figure 2C), or a length effect due to topoisomerase deficiency is masked by a much stronger effect from reduced initiation. Our data are consistent with the earlier observed length independency of transcription and intragenic RNA polymerase II binding in budding yeast *top1Δtop2ts* cells [9], [22], [46].

### Transcriptional activation of highly regulated, chromatin-dependent genes requires topoisomerase-mediated DNA relaxation

Based on our genome-wide studies a pattern emerges for genes, which are strongly dependent on topoisomerases. This gene group is enriched for genes with high responsiveness to environmental changes, a TATA box in their promoter, SAGA complex dependency, high transcriptional plasticity, sensitivity to chromatin regulation, and specific nucleosome architecture in the promoter (Figure 3; Figures S3, S4, S5; and Table S2). In summary, the analyses point toward a highly regulated mode of transcriptional activation for this group of genes.

How can an effect on repressible/inducible genes be unraveled from microarray experiments performed with cells grown exclusively in rich media (YPD)? For the majority of these genes, rich media will neither give complete repression nor complete activation of transcription. Rather, a subpopulation of cells will have a given gene in an active form, while the remaining cells will have the gene in a repressed form. We interpret a drop in transcript levels between wild-type cells and *top1Δtop2ts* cells in YPD media to reflect an inhibition of activation in these subpopulations due to topoisomerase deficiency. We therefore conclude that genes with high transcriptional plasticity are scored as de-regulated genes in YPD because topoisomerase-deficiency will perturb the periodic activation and/or repression of transcription of these genes.

The observed enrichment of stress responsive genes among the up-regulated genes in *top1Δtop2ts* cells (Table S1) could indicate that lack of topoisomerases leads to a general stress response. This could be the case if central players in the common response to environmental changes like activators or repressors are de-regulated by the lack of the enzymes or indirectly by the slow growth of *top1Δtop2ts* cells [47]. Arguing strongly against this, is that our Gene Ontology analysis did not reveal any enrichment of transcriptional activators and repressors including those involved in the regulation of stress responsive genes (Table S1). Pertinent to this discussion, stress responsive genes are primarily found in telomere proximal regions [48], [49], but the genes affected in *top1Δtop2ts* are not biased towards these regions (Table S2).

In further support of a role of topoisomerases for regulated gene transcription we show that the enzymes stimulate transcriptional activation of twelve different inducible genes, including the *PHO* genes, the *GAL* genes, *ADH2*, and *INO1* (Figure 4). Attention has been drawn to some of these genes in earlier studies of topoisomerases. Thus, in a study of the *PHO5* promoter nucleosome positioning it was noticed that *PHO5* transcription was inhibited in the absence of topoisomerases [50]. Furthermore, *ADH2* has been reported to be regulated by Top1p, which was suggested to repress its transcription by relaxation of negative DNA superhelicity [51]. Our finding that efficient *GAL1* activation is dependent on topoisomerases is in contrast to earlier observations with endogenous *GAL1* and a plasmid-borne *GAL1*-lacZ construct [1], [6], where high levels of *GAL1* transcription was seen in the absence of topoisomerases. However, in a separate study, transcription of *GAL1* was found to be strongly inhibited upon expression of *E. coli* TopA in the absence of yeast topoisomerases [8] in support of a role of these enzymes in *GAL1* transcription. We have observed similar topoisomerase dependency in two different yeast strains, indicating that strain background and variable *top2ts* mutations do not account for the observed inconsistency (data not shown). As we have used G1 arrested cells to exclude replication-associated effects in *top1Δtop2ts* rather than exponentially growing cells, it remains possible that different supercoiling levels exist in different phases of the cell cycle, which may underlie the discrepancy between ours and earlier studies concerning the dependency of *GAL1* transcription on topoisomerases.

Use of G1 arrested cells could also affect the studies of *PHO5* induction, as arrested cells have been demonstrated to accumulate polyphosphate, a vacuolar Pi reserve, which will influence the rate of *PHO5* induction [44], [45]. It could thus be speculated that this reserve is responsible for the lack of *PHO5* induction observed in the *top1Δtop2ts* cells, as these cells, due to their decreased metabolic activity, would be expected to need more time to consume the Pi reserve relative to wild-type cells. To exclude this possibility we deleted *VTC1* and *VTC4* in wild-type and *top1Δtop2ts*, as these genes are the two main genes required for polyphosphate synthesis. As seen in Figure S8, like in *top1Δtop2ts* cells, induction of *PHO5* still does not take place in *vtc1Δtop1Δtop2ts* and *vtc4Δtop1Δtop2ts* cells, confirming that it is the lack of topoisomerases and not excessive Pi reserves, which causes inhibition of *PHO5* induction. This was further demonstrated by studying *PHO5* induction using exponentially growing cells rather than G1 arrested cells (Figure S9).

It is not yet clear how topoisomerases exert their function during transcription of highly regulated genes. Given that topoisomerase deficiency results in both up- and down-regulations (with the vast majority of genes being down-regulated, Figure 1), both stimulatory and repressive activities are potentially affected during transcription of these genes in *top1Δtop2ts*. In line with this, a highly regulated transcription pattern is known to be orchestrated by transactions between specific activators/repressors and their DNA binding sites, as well as by chromatin structure [32], [33], [52].

Interestingly, in the case of the *PHO5* gene, topoisomerases are required for binding of the Pho4p transcription factor, which is critical for subsequent promoter nucleosome removal and transcriptional induction (Figure 7). *PHO5* thus provides an example, where topoisomerase activity is required for a step upstream of the engagement of polymerases. Since we observe *PHO5* induction, although with a kinetic delay, in *top1Δ* and *top2ts* single mutants at the restrictive temperature (Figure 6A), as well as in *top1Δtop2ts* at the permissive temperature (data not shown), we find it unlikely that either one of these enzymes play a more specific role during transcription initiation of *PHO5*. Rather, our data suggest that it is lack of their redundant DNA relaxation activity that influences *PHO5* transcription. In support of this, we find that *E. coli* TopA-mediated changes in global supercoiling levels in wild-type cells result in altered transcriptional output from *PHO5* (Figure 6B).

We reason that indirect effects caused by a potential transcriptional de-regulation of co-factors in the *PHO5* induction pathway in *top1Δtop2ts* is implausible, since Pho4p enters the nucleus (Figure 7C), and only minor expression changes were seen with transcription factors involved in *PHO5* transcription (data not shown). Furthermore, we expect most of these effects to result in delayed *PHO5* induction kinetics, as seen for the topoisomerase single mutants, rather than a total inhibition [42], [43].

Taken together, our investigations suggest that DNA topoisomerases are required to maintain the genome in a state competent for transcription initiation. Top1p and Top2p seem to exert this role by a mutually redundant relaxation of DNA supercoils, thus influencing highly transcribed genes and highly regulated, chromatin-dependent genes. Any imbalance in net DNA superhelicity, which likely appears in *top1Δtop2ts*, may have profound effects on chromatin-regulated promoters. Topoisomerase deficiency may have pleiotropic effects affecting polymerase recruitment [18], [22], nucleosome assembly/disassembly equilibrium [14], [53] or steps upstream to these activities as is the case with *PHO5*, where binding of a transcription factor is inhibited. The different scenarios are not mutually exclusive, and DNA topoisomerases most likely function at numerous levels to influence DNA superhelicity for maintenance of transcriptional competency.

## Materials and Methods

### Yeast strains and growth conditions

All *S. cerevisiae* strains are derivatives of W303a, and the associated manipulations for obtaining derivate strains are according to standard genetic techniques. For microarray analysis, yeast strains were grown to exponential phase at 25°C in YPD and further grown for 90 min at 25°C in YPD with α-factor (Lipal Biochem, Zürich, Switzerland) to synchronize cells in G1. Cultures were then placed at 37°C for another 90 min for conditional inhibition of Top2p, where more α-factor was added to keep cells in G1. Cultures were adjusted, so that an equal number of cells could be used for all yeast strains (6×10^7^ cells). Finally, cells were harvested by centrifugation at 37°C. For each sample, aliquots were collected for fluorescence-activated cell sorting analysis as previously described [54] to ensure successful and persistent cell cycle arrest (data not shown). Three independent sets of experiments were performed to obtain triplicate biological measurements. As the great majority of transcripts in yeast have short decay rates [55], 90 min of Top2p inactivation was chosen before RNA extraction to ensure turnover of transcripts produced prior to conditional inhibition of Top2p. For analyses of gene-activation in four different inducible gene systems, cells were prepared as for the microarray analysis, except that cells were grown under individual repressive conditions and Top2p was inhibited at 37°C for 15 min prior to transfer of cells to the respective inducible conditions (see Text S1 for composition of the various growth media). For the *PHO5* activation experiments, cells were prepared as for the microarray analysis, but instead of YPD they were cultured in high phosphate medium (yeast nitrogen base w/o phosphate and amino acids from ForMedium, Norfolk, UK). Glucose was added to 2%, amino acids were added to standard concentrations, and KH_2_PO_4_ was added to a concentration of 15 mM. After cell cycle arrest in G1 and conditional inhibition of Top2p, cells were shifted to phosphate-free medium (as above, but without addition of KH_2_PO_4_ and supplemented with 7.35 mM KCl) for induction of *PHO5*. The fold increase observed in *PHO5* mRNA levels, when cells are kept in phosphate-free medium for 180 minutes varies from experiment to experiment in the range from 50 fold to 350 fold. For this reason we show *PHO5* inductions as percentages of maximum transcript accumulated in the wild-type strain. For transcriptional repression of *PHO5*, cells were first cultured in phosphate-free medium at 25°C for 4 h to obtain high *PHO5* transcription, where α-factor was added after 2,5 h. The temperature was then increased to 37°C to inhibit Top2p, and after 15 min at 37°C, cells were transferred to high phosphate conditions (15 mM KH_2_PO_4_) for *PHO5* repression. See Table S3 for a list of strains used in this study.

### RNA preparations, microarray experiments, and normalization

For the microarray experiments total RNA was initially prepared by acid phenol extraction. Immediately prior to RNA extraction, external spike-in Poly-A RNA's (Affymetrix, Santa Clara, CA) were added to an equal number of cells from all four yeast strains to enable external normalization. High-quality RNA was obtained by further purification of phenol-extracted RNA on RNeasy columns (Qiagen, Valencia, CA) according to manufacturer's directions. RNA quality was assessed by gel electrophoresis and spectrophotometry (GeneQuant II, Pharmacia Biotech). Gene expression profiling was performed using Affymetrix Yeast Genome 2.0 GeneChip oligonucleotide arrays essentially according to Affymetrix protocols. Normalization procedure and data processing can be viewed in detail in Text S1.

### Analysis of genome-wide transcriptional effects

To estimate global mRNA changes we calculated and compared the total intensity from all detectable probe sets on the mutant and wild-type arrays. For correlation to wild-type transcription levels, all mRNA abundances were averaged across biological triplicates (microarray signal values). Arbitrary transcriptional activities were calculated by dividing average expression levels in the triplicate wild-type arrays by genome-wide mRNA half-life data [55] (URL: http://www-genome.stanford.edu/turnover/), as described by Schreiber and colleagues [26]. These measures were median-normalized for presentation in Figure 2. We collected transcript lengths from the transcription map generated by David et al. [27].

### Analysis of transcriptional plasticity and chromatin regulation

Measures of transcriptional plasticity for every gene were obtained from Barkai and co-workers [32]. Measures of sensitivity to chromatin regulation were derived by Choi and Kim [34] (gathered from URL: http://www.nature.com/ng/journal/v41/n4/suppinfo/ng.319_S1.html). Analysis of nucleosome occupancy was performed with the use of a recent map of nucleosome positions in *S. cerevisiae* [35] (URL: http://chemogenomics.stanford.edu/supplements/03nuc/). The data on nucleosome positions aligned according to transcription start site were used.

### Chromatin immunoprecipitation and qPCR

For analysis of transcription levels, cells were grown as described above, and samples (∼10^8^ cells) were taken at the indicated time points. RNA was purified as for the microarray analysis followed by DNase I treatment, and cDNA was made by SuperScript II RT-PCR (Invitrogen, Carlsbad, CA) using oligo dT primer. Real-time PCR was performed with DYNAmo SyBR Green qPCR kit (Finnzymes, Vantaa, Finland) and used to quantify mRNA levels, using a Stratagene MX3000 (Agilent, Santa Clara, CA). For each yeast strain, Ct-values from triplicate qPCR amplifications were averaged across three independent measurements. ChIP was performed on 2.5×10^8^ cells as described previously [54] with minor modifications. Histone H3 was precipitated with monoclonal antibodies recognizing the C-terminal tail (ab1791 available from Abcam, Cambridge, UK) and Pho4-13xcMyc was precipitated using a monoclonal antibody (Santa Cruz Biotech, Santa Cruz, CA). For ChIP of Pho4-13xcMyc, cell extract was incubated with beads coupled with antibody overnight instead of 2 h. For H3 ChIP, fold increase was calculated between antibody-coupled Dynabeads (IP) and BSA-coated Dynabeads (background) and normalized to the fold increase from an intra-genic sequence in a gene (*YOL151W*) not affected by topoisomerase activity as assessed by qPCR (data not shown), and the 0 min time point was set to 1. Normalizing to a telomeric locus (*TEL06R*) gave similar results. Pho4-13xcMyc ChIP was calculated in the same way, but using the *GAL1/10* promoter region as control region. Primer sequences are listed in Table S4.

### Fluorescence microscopy

Wild-type and *top1Δtop2ts* cells were treated as for the *PHO5* induction experiments, and fluorescence microscopy was performed as described previously [54].

### Gene Expression Omnibus accession numbers

The gene expression data have been deposited in the NCBI Gene Expression Omnibus database with accession number GSE22809.

## Supporting Information

Figure S1 Topoisomerase dependency correlates with RNA polymerase II binding in ORF's. *top1Δtop2ts* gene expression changes (between mutant and wild-type) plotted against average RNA polymerase II (RNA pol II) occupancy in open reading frames as a 200 gene moving average. Data on RNA polymerase II occupancy were gathered from [56]. Pearson correlation is −0.16.
(EPS)

Figure S2 Association between gene expression level and topoisomerase dependency for functionally classified gene groups. Gene groups based on the most general and overall functional classifications were retrieved from the MIPS FunCat database [57]. For each functional group, the average mRNA abundance of genes within the group and the fraction of de-regulated genes in the *top1Δtop2ts* microarray data set (% genes) were calculated. De-regulated genes were defined as being up- or down-regulated with +0.5 and −1 cutoffs in the signal log2 ratio between mutant and wild-type, respectively. *Upper histogram*, the average mRNA abundance for genes in each of the functional gene groups (groups 1–15) was normalized to the genome-wide average (arbitrarily set to 1). *Lower histogram*, the fraction of de-regulated genes in *top1Δtop2ts* is shown for each functional gene group. The MIPS functional gene groups and their number of genes are listed to the right. The functional gene groups with highest and lowest expression are indicated by orange and blue colors, respectively. For comparison, the TATA-less and TATA-containing gene groups [31] are included (groups 16 and 17, respectively).
(EPS)

Figure S3 Transcriptional plasticity correlates with topoisomerase dependency independent of gene expression levels. The analysis presented in Figure 3A was repeated with groups of genes based on wild-type transcription levels. Thus, all genes were divided into quartiles with decreasing mRNA abundances, where the 1st quartile represents the gene group with the highest mRNA abundance. For each of these gene groups the transcriptional plasticity [32] was plotted against *top1Δtop2ts* gene expression changes as a 200 gene moving average.
(EPS)

Figure S4 Expression changes in *top1Δtop2ts* correlate with expression changes obtained from yeast strains with perturbation of different chromatin factors. Gene expression changes in *top1Δtop2ts* (SLR, signal log2 ratio between mutant and wild-type) are plotted as a function of gene expression changes (SLR, signal log2 ratio) generated from perturbation of different chromatin regulators. (A) *spt6ts* [58], (B) *spt16ts* [58], (C) *taf1-2ts spt3(E240K)* [30], (D) *gcn5(KQL)* [30], (E) *paf1Δ* [59], (F) histone H4 depletion (4 h timepoint) [49], (G) histone H3Δ1-18 [60], and (H) *top1Δtop2ts + TopA* vs. *top1Δ* (120 min time point and all expression changes divided by 2, because all transcript levels are approximately one log2 higher than the real value, as described by the authors) [9]. *R* denotes the Pearson correlation coefficient, and the associated correlation *P*-value (*P*) was calculated by permutation testing. Genes in the lower 0.05 and upper 0.95 percentiles for expression changes were specified as the most de-regulated genes in each dataset, and *P_o_* denotes the *P*-value of the overlap between de-regulated gene sets from the chromatin regulators and *top1Δtop2ts*, using a hypergeometric test.
(TIF)

Figure S5 Confidence intervals for nucleosome occupancy in promoter regions of genes from different gene groups. Profiles of the average nucleosome-binding pattern [35] in the nucleosome-free region (NFR) proximal to transcription start sites (TSS) is shown for groups of the 100 most unaffected genes (A), the 100 most up-regulated genes (B), and the 100 most down-regulated genes (C) in *top1Δtop2ts*. Error bars represent 95% confidence intervals.
(EPS)

Figure S6 Transcription of non-inducible genes under inducible conditions in wild-type and *top1Δtop2ts* cells. Time course experiments of control gene expression in wild-type and *top1Δtop2ts* cells under inducible conditions, where the experimental setup was as shown in Figure 4A. mRNA levels of three housekeeping genes (*ESC1*, *ACT1* and *GAPDH*) were quantified by qPCR at the indicated time points after transfer of cells to either phosphate- (*upper panel*), galactose- (*middle panel*), or glucose-inducible conditions (*lower panel*). mRNA levels were normalized to the mRNA level obtained in the wild-type at the 0 min time point (set to 100%). Averages from two individual experiments are shown with error bars representing ± one standard deviation.
(EPS)

Figure S7 Pho4-13xcMyc cells display wild-type *PHO5* induction kinetics. Time-course experiments of *PHO5* transcription in wild-type and Pho4-13xcMyc cells. The experimental setup was as described for Figure 5A. The quantified *PHO5* mRNA levels were normalized to the wild-type mRNA level at the 180 min time point (set to 100%). Averages from two individual experiments are shown with error bars representing ± one standard deviation. Number indicates the mean fold increase in *PHO5* mRNA levels at the latest time point.
(EPS)

Figure S8 Lack of *PHO5* induction in topoisomerase deficient cells is not caused by internal polyphosphate storages. Time-course experiment of *PHO5* transcription in *vtc1Δ* and *vtc1Δtop1Δtop2ts* cells (*left panel*), *vtc4Δ* and *vtc4Δtop1Δtop2ts* cells (*middle panel*) or wild-type, *vtc1Δ* and *vtc4Δ* cells (*right panel*) after transfer from high phosphate to phosphate-free conditions, where the quantified *PHO5* mRNA levels were normalized to the *vtc1Δ* level at the 180 min time point (set to 100%), the *vtc4Δ* level at the 180 min time point (set to 100%), or the wild-type level at the 180 min time point (set to 100%), respectively. Averages from two individual experiments are shown with error bars representing ± one standard deviation. Numbers indicate the mean fold increase in *PHO5* mRNA levels in the indicated strains at the latest time point. The experimental setup was as described for Figure 4A, using the conditions for the phosphate-responsive genes. The comparison between wild-type, *vtc1Δ* and *vtc4Δ* cells (*right panel*) demonstrates that *vtc1Δ* and *vtc4Δ* cells show more rapid and higher maximum activation of *PHO5* relative to wild-type cells in agreement with previous studies [44].
(EPS)

Figure S9 Lack of *PHO5* induction in topoisomerase deficient cells is not caused by G1 cell cycle arrest. *Upper panel*, experimental setup, where +P_i_ and −P_i_ indicate high and no phosphate, respectively. *Lower panel*, time-course experiment of *PHO5* transcription in asynchronous growing wild-type and *top1Δtop2ts* cells after transfer from high phosphate to phosphate-free conditions. The quantified *PHO5* mRNA level was normalized to the wild-type level at the 180 min time point (set to 100%). Average from three individual experiments is shown with error bars representing ± one standard deviation. Number indicates the mean fold increase in *PHO5* mRNA levels in wild-type cells at the latest time point.
(EPS)

Table S1 Gene Ontology analysis of genes affected 2-fold or more in *top1Δtop2ts*. Using Funspec software [61], a stringent P-value cutoff of 1.00E-03 was used. k: number of genes from the input cluster in a given category. f: total number of genes in a given category.
(EPS)

Table S2 Characterization of genes de-regulated 2-fold or more in *top1Δtop2ts*. ^a^Gene sets were tested for overlap with genes regulated by the stress-related SAGA (Spt-Ada-Gcn5-Acetyltransferase) complex [30]. The hypergeometric distribution was used to calculate overlap probabilities (*P*). ^b^Gene sets were tested for overlap with TATA-box containing genes [31]. The hypergeometric distribution was used to calculate overlap probabilities (*P*). ^c^A measure for responsiveness to environmental changes was derived for every gene using the Gasch dataset [28], which measures expression ratios across 173 conditions of diverse environmental changes. This measure was calculated as the average of the squared log2 expression ratio from all 173 microarray profiles on environmental change, thus calculating the dynamic range of expression levels under different conditions [32]. The squared expression ratios were set to a mean of 0 and a standard deviation of 1. The responsiveness to environmental changes for the groups of 2-fold or more up- and down-regulated genes, respectively, and the rest of the genome were compared using the t-test. *P* values for higher responsiveness to environmental changes are reported. ^d^Distance to the closest telomere was compared to the rest of the genes by the t-test.
(EPS)

Table S3 *S. cerevisiae* strains used in this study.
(EPS)

Table S4 Primers used in qPCR for quantification of ChIP levels and gene expression levels.
(EPS)

Text S1 Supplementary Materials and Methods.
(DOCX)
