# Supplementary figures and images for: DNA Topoisomerases Maintain Promoters in a State Competent for Transcriptional Activation in Saccharomyces cerevisiae
Source: PLoS Genet. 2012 Dec 20;8(12):e1003128. doi: 10.1371/journal.pgen.1003128 (PMC3527272; doi:10.1371/journal.pgen.1003128)

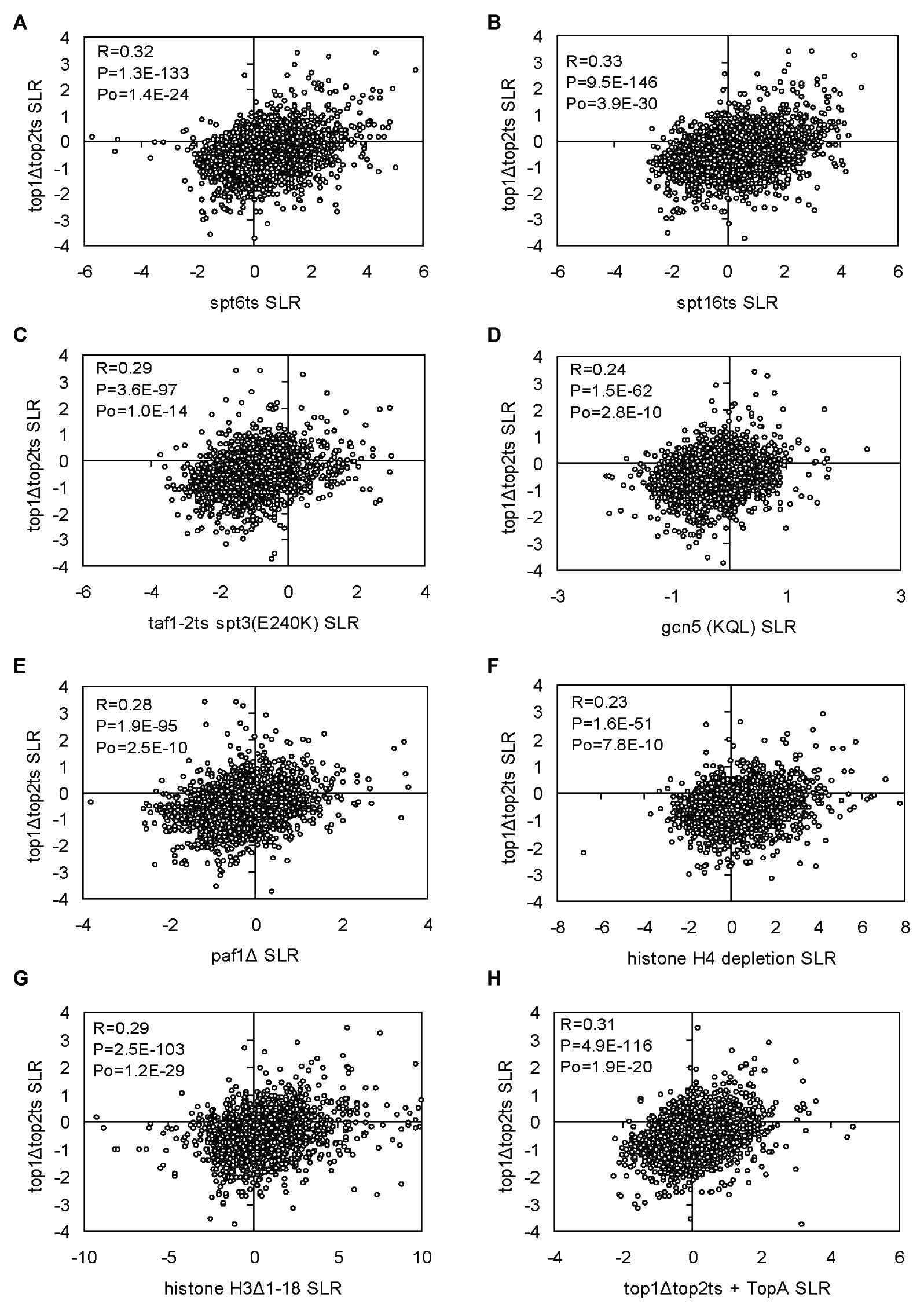

Supplement: Figure S4 — Expression changes in top1Δtop2ts correlate with expression changes obtained from yeast strains with perturbation of different chromatin factors. Gene expression changes in top1Δtop2ts (SLR, signal log2 ratio between mutant and wild-type) are plotted as a function of gene expression changes (SLR, signal log2 ratio) generated from perturbation of different chromatin regulators. (A) spt6ts [58], (B) spt16ts [58], (C) taf1-2ts spt3(E240K) [30], (D) gcn5(KQL) [30], (E) paf1Δ [59], (F) histone H4 depletion (4 h timepoint) [49], (G) histone H3Δ1-18 [60], and (H) top1Δtop2ts + TopA vs. top1Δ (120 min time point and all expression changes divided by 2, because all transcript levels are approximately one log2 higher than the real value, as described by the authors) [9]. R denotes the Pearson correlation coefficient, and the associated correlation P-value (P) was calculated by permutation testing. Genes in the lower 0.05 and upper 0.95 percentiles for expression changes were specified as the most de-regulated genes in each dataset, and Po denotes the P-value of the overlap between de-regulated gene sets from the chromatin regulators and top1Δtop2ts, using a hypergeometric test. (TIF) [file pgen.1003128.s004.tif]
