# Supplementary material for: DNA Topoisomerases Maintain Promoters in a State Competent for Transcriptional Activation in Saccharomyces cerevisiae
Source: PLoS Genet. 2012 Dec 20;8(12):e1003128. doi: 10.1371/journal.pgen.1003128 (PMC3527272; doi:10.1371/journal.pgen.1003128)
Supplement: Text S1 — Supplementary Materials and Methods. (DOCX) [file pgen.1003128.s014.docx]

**Text S1**

**Microarray analyses and normalization**

Gene expression profiling was performed using Affymetrix Yeast Genome 2.0 GeneChip oligonucleotide arrays (Affymetrix, Santa Clara, CA) essentially according to Affymetrix protocols. Data processing and external normalization was carried out with Affymetrix GeneChip Operating software. For comparison of mutant and wild-type counterparts, we pre-filtered the expression profiles, so that a given gene was scored only if a “Present” call had been assigned by the MAS5 algorithm in each of the triplicate expression profiles. For all genes scored, the mutant to wild-type signal fold change was calculated, and the average fold change of the three independent experiments was used. A few genes were measured by several probe sets, and these replicate measures were averaged. All fold changes were log2 transformed. The completely filtered *top1Δtop2ts* dataset contains 5,613 protein-coding genes (4,754 verified, 830 uncharacterized and 20 dubious, *Saccharomyces* Genome Database, <http://www.yeastgenome.org/>, annotated in April 2010).

**Growth conditions for analyses of transcriptional activation of genes in different inducible gene systems**

Wild-type and *top1Δtop2ts* cells were prepared in parallel as for the microarray analyses with the following differences: Cells were cultured in the respective repressive media (see below) at 25ºC to a density of ~10^7^ cells/ml, G1 cell cycle arrested with alpha-factor (Lipal Biochem, Zürich, Switzerland), and Top2p was inhibited for 15 min at 37ºC under repressive conditions. Next, cells were washed twice, shifted to the respective inducible conditions in pre-warmed media, and incubated at 37ºC.

For induction of the galactose-responsive genes, cells were not washed for shift of medium, but galactose was simply added to the growth medium (see below). Samples with equal amounts of cells (~10^8^ cells) were taken at the different time points as indicated in Figure 4.

For activation of phosphate-responsive genes (*PHO5, PHO8, VTC1, VTC3*), cells were grown in repressive high phosphate medium (yeast nitrogen base w/o phosphate and amino acids from ForMedium, Norfolk, UK). Glucose was added to 2%, amino acids were added to standard concentrations, and KH_2_PO_4_ was added to a concentration of 15 mM. For induction, cells were shifted to phosphate-free medium: As above, but without KH_2_PO_4_ and supplemented with 7.35 mM KCl.

For activation of galactose-responsive genes (*GAL1, GAL2, GAL7, GAL10*), cells were grown in repressive YP medium with 2% raffinose, and galactose was added to a final concentration of 2% for induction.

For activation of glucose-repressed genes (*ADH2, ADY2, YAT1*), cells were grown in repressive SC medium with 8% glucose, and shifted to SC medium with 0.1% glucose and 3% ethanol for induction.

For activation of *INO1*, cells were grown in repressive high inositol medium: yeast nitrogen base w/o amino acids and w/o inositol from ForMedium (ForMedium, Norfolk, UK), supplemented with 100 µM *myo*-inositol and 1 µM choline chloride, and amino acids and glucose were added to standard concentrations. For induction, cells were shifted to inositol-free medium: As above, but without *myo*-inositol and choline chloride.

**RNA extraction and qPCR**

For analysis of *PHO5* transcript levels during G1 arrest but in the absence of phosphate storages (Figure S8), wild-type, *vtc1∆*, *vtc4∆*, *top1∆top2ts*, *vtc1∆top1∆top2ts*, and *vtc4∆top1∆top2ts* cells were grown as described above, and samples (~2x10^7^ cells) were taken at the indicated time points. For asynchronously growing cells (Figure S9), yeast strains were grown to exponential phase at 25ºC in high phosphate medium, washed in sterile H_2_O and transferred to phosphate-free medium pre-heated to 37ºC, and samples (~2x10^7^ cells) were collected at the indicated time points. RNA was extracted using RNeasy kit from Invitrogen according to the manufacturer’s direction. DNaseI treatment, cDNA synthesis, and qPCR were performed as described in Materials and Methods except that for each strain, *PHO5* mRNA levels were normalized using the mRNA levels of the housekeeping genes *GAPDH (TDH1)* and *ACT1* instead of spike-ins.
